# Identification of PP2A-B55 targets uncovers regulation of emerin during nuclear envelope reassembly in *Drosophila*

**DOI:** 10.1098/rsob.230104

**Published:** 2023-07-19

**Authors:** Virginie Emond-Fraser, Myreille Larouche, Peter Kubiniok, Éric Bonneil, Jingjing Li, Mohammed Bourouh, Laura Frizzi, Pierre Thibault, Vincent Archambault

**Affiliations:** ^1^ Institute for Research in Immunology and Cancer, Université de Montréal, Montréal, H3T 1J4, Quebec, Canada; ^2^ Département de biochimie et médecine moléculaire, Université de Montréal, Montréal, H3T 1J4, Quebec, Canada; ^3^ Département de chimie, Université de Montréal, Montréal, H3T 1J4, Quebec, Canada

**Keywords:** nuclear envelope, mitosis, emerin, otefin, PP2A, BAF

## Abstract

Mitotic exit requires the dephosphorylation of many proteins whose phosphorylation was needed for mitosis. Protein phosphatase 2A with its B55 regulatory subunit (PP2A-B55) promotes this transition. However, the events and substrates that it regulates are incompletely understood. We used proteomic approaches in *Drosophila* to identify proteins that interact with and are dephosphorylated by PP2A-B55. Among several candidates, we identified emerin (otefin in *Drosophila*). Emerin resides in the inner nuclear membrane and interacts with the DNA-binding protein barrier-to-autointegration factor (BAF) via a LEM domain. We found that the phosphorylation of emerin at Ser50 and Ser54 near its LEM domain negatively regulates its association with BAF, lamin and additional emerin in mitosis. We show that dephosphorylation of emerin at these sites by PP2A-B55 determines the timing of nuclear envelope reformation. Genetic experiments indicate that this regulation is required during embryonic development. Phosphoregulation of the emerin–BAF complex formation by PP2A-B55 appears as a key event of mitotic exit that is likely conserved across species.

## Graphical abstract

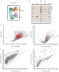

## 1. Introduction

The mitotic cycle is largely driven by kinases and phosphatases that control reversible phosphorylation on various effector proteins to modify their activities [1]. During mitotic entry, phosphorylation by cyclin-dependent kinases (CDKs), Polo-like kinase 1 and aurora kinases are largely responsible for the induction of nuclear envelope breakdown (NEBD), chromosome condensation, spindle assembly and other cellular events that prepare the cell for the segregation of chromosomes [2–4]. Mitotic exit is triggered by the anaphase-promoting complex/cyclosome that ubiquitinates mitotic cyclins and securin, targeting them for degradation by the proteasome [5]. As a result, sister chromatids are separated, allowing their segregation in anaphase. Quickly after, the nuclear envelope starts to reassemble from the endoplasmic reticulum (ER) around segregated chromosomes that begin to decondense [6,7]. In addition, the spindle is disassembled, and kinetochores and centrosomes are partly dismantled [1]. Several proteins become dephosphorylated in this transition [8,9]. The precise contributions of these dephosphorylation events to mitotic exit and the specific roles of phosphatases in this process are incompletely understood.

**Figure 1.**
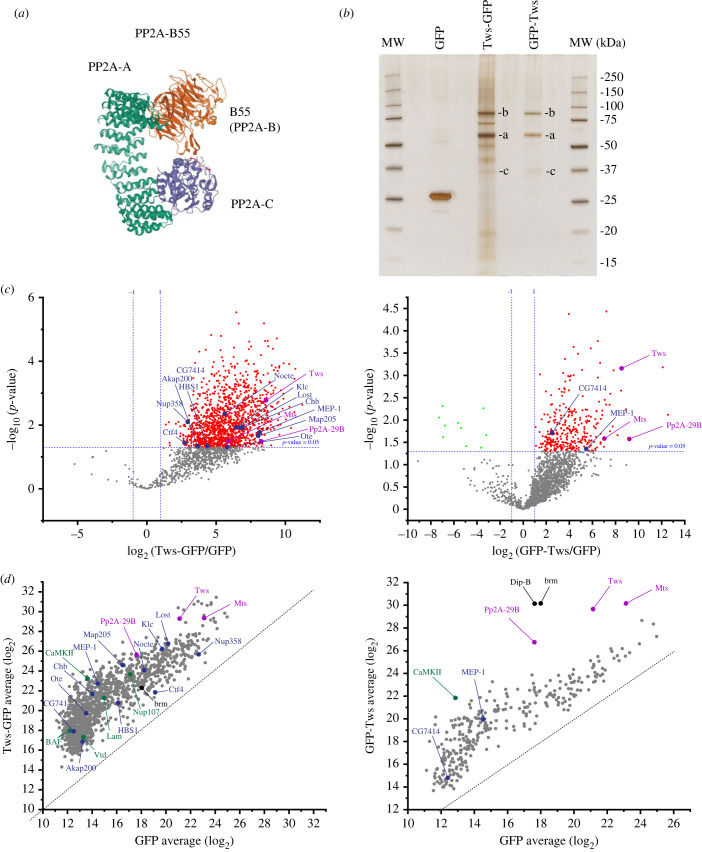
Identification of PP2A-Tws interactors. (*a*) Crystal structure of human PP2A-B55 holoenzyme (PDB 3BW8). The structural (PP2A-A, green), catalytic (PP2A-C, purple) and regulatory (PP2A-B, orange) subunits are indicated. (*b*) Purification of PP2A-Tws complexes from *Drosophila* embryos. Zero to 2 h-old embryos expressing GFP, Tws-GFP or GFP-Tws were submitted to GFP affinity purifications. The gel shows 5% of the total purification products on a silver-stained 4–12% acrylamide SDS-PAGE gel. The rest of the samples were submitted to mass spectrometric analyses. The positions of holoenzyme subunits are indicated as: a = PP2A-29B (PP2A-A, 65 kDa predicted); b = Tws-GFP/GFP-Tws (PP2A-B, 80 kDa predicted); c = Mts (PP2A-C, 35 kDa predicted). MW: Molecular weight. (*c*) Identification of proteins significantly enriched with Tws-GFP or GFP-Tws compared to GFP alone. Data points are from three independent experiments. (*d*) Scatter plots showing abundances of proteins significantly enriched with Tws-GFP or GFP-Tws compared to GFP alone (only proteins in red from c)). For (*c*) and (*d*): purple names: PP2A-Tws holoenzyme subunits; green names: previously reported PP2A-Tws substrates; blue names: proteins identified as likely targets of PP2A-Tws in figure 2.

The protein phosphatases 1 (PP1) and 2A (PP2A) are required for several aspects of mitotic progression [9–11]. PP2A forms trimers comprising a catalytic subunit (PP2A-C), a structural subunit (PP2A-A) and a regulatory subunit (PP2A-B) (figure 1*a*) [12]. The B55 subunit provides substrate specificity to PP2A in part via surface-exposed amino acid residues that bind targets transiently [10,13,14]. PP2A-B55 tends to efficiently dephosphorylate sites immediately preceding a proline residue, which include CDK sites [15–20]. PP2A-B55 also dephosphorylates phospho-threonines more efficiently than phospho-serines [20,21]. Humans possess four B55 subunit genes, with B55*α* and B55*δ* most ubiquitously expressed [22]. *Drosophila* has only one B55 gene, named *twins* (*tws*) or *abnormal anaphase resolution* (*aar*), and its mutation or knockdown results in mitotic defects [23–25].

**Figure 2.**
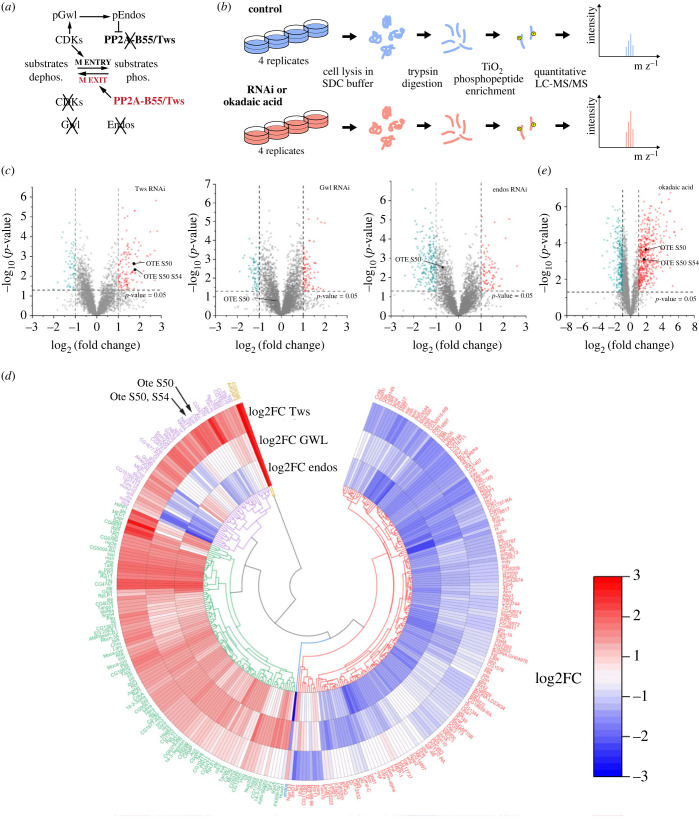
Identification of PP2A-Tws-dependent phosphoproteins. (*a*) Wiring diagram of PP2A-B55/Tws function and regulation in the mitotic cycle. See Introduction for details. (*b*) Experimental scheme for the identification of PP2A-Tws-dependent phosphorylation sites. (*c*) Identification of phosphopeptides of which the abundance depends on Tws, Gwl or Endos. Volcano plots indicate average fold-change values of peptides from the indicated RNAi samples compared to non-target RNAi control samples analysed in quadruplicate. Significantly enriched (top-right, red) or depleted (top-left, teal) peptides are displayed. Otefin peptides phosphorylates at Ser50 and/or Ser54 are shown. (*d*) Radial heat map for differentially regulated phosphopeptides identified in (*c*). Colour scale indicates log_2_ fold change. (*e*) Identification of phosphopeptides sensitive to OA treatment. The volcano plot indicates average fold-change values of peptides from OA-treated samples compared to DMSO control samples analysed in quarduplicate. Significantly enriched (top-right, red) or depleted (top-left, teal) peptides are displayed. Otefin peptides phosphorylated at Ser50 and/or Ser54 are shown.

PP2A-B55 is inactivated during mitotic entry [18]. Cyclin B-CDK1 activates the Greatwall (Gwl)/MASTL kinase that then phosphorylates endosulfine proteins (Endos in *Drosophila*), turning them into selective inhibitors of PP2A-B55 [26–30] (figure 2*a*). Endosulfines act as inhibitors because they are substrates of PP2A-B55 with a relatively high affinity for the enzyme and a very low dephosphorylation rate [31]. In late mitosis, when Cyclin B is degraded, Gwl is inactivated by PP1 and endosulfines are ultimately dephosphorylated by PP2A-B55 which thereby becomes reactivated [31–33]. This system generates a delay between anaphase onset and later events of mitotic exit that depend on PP2A-B55 [34].

Efforts to identify PP2A-B55/Tws functions and substrates in the cell cycle have used different strategies. A live imaging screen found PP2A-B55*α* to be required for several events of mitotic exit including nuclear envelope reformation (NER), chromosome decondensation and spindle disassembly [35]. A kinetic phosphoproteomic study identified several PP2A-B55-dependent substrates to propose motif constraints in PP2A-B55 target sites [14]. A role for PP2A-B55 in promoting NER was also uncovered by this study, with several nucleoporins being identified as targets. Phosphoproteomic characterization of Gwl KO cells also revealed roles of PP2A-B55 in the regulation of the spindle assembly checkpoint and in the maintenance of genome integrity [36,37]. Phosphoproteomic studies in the yeast *S. cerevisiae* have revealed several substrates of PP2A-Cdc55 (the B55 orthologue) in the regulation of mitosis [38–40]. However, the regulation and functions of this enzyme in the cell cycle appear to differ considerably between budding yeast and animals [41]. Moreover, the nuclear envelope does not break down during mitosis in yeast [42].

We previously took a genetic approach to search for functions and targets of PP2A-Tws in mitosis. We found that PP2A-Tws promotes NER in mitosis and in meiosis [43]. This occurs in part through its dephosphorylation of barrier-to-autointegration factor (BAF), a protein that links chromosomes, nuclear envelope proteins and the lamina in interphase [44]. BAF directly binds DNA and the LEM (Lem2, emerin, MAN1) domain contained in several proteins of the inner nuclear membrane [6,44,45]. In mitosis, BAF phosphorylation in its N-terminus by VRK1 (NHK-1 in *Drosophila*) induces its dissociation from DNA and decreases its affinity for LEM domains [46,47]. During mitotic exit, dephosphorylation of BAF by PP2A results in BAF recruitment to segregated chromosomes [43,48]. There, BAF serves as a platform for NER by recruiting LEM-domain proteins [6,44,45]. PP2A-B55/Tws could promote NER by dephosphorylating several other proteins [8].

Here, we used proteomic approaches to identify targets of PP2A-Tws during mitotic exit in *Drosophila*. We identified several specific PP2A-Tws interactors and PP2A-Tws-dependent phosphorylation sites. Otefin (also known as emerin from its name in humans), a LEM-domain protein that interacts with BAF, emerged as an important target [49,50]. In humans, mutations in the emerin gene cause Emery–Dreifuss muscular dystrophy [51,52]. Work in *Xenopus* egg extracts had suggested that emerin and MAN1 phosphorylation in mitosis decreases their ability to bind BAF [53,54]. However, the precise mechanism, the phosphorylation sites and the kinase(s) and phosphatase(s) responsible for this potential regulatory mechanism remained unknown. Here we show that otefin dephosphorylation in a PP2A-Tws-dependent manner promotes the formation of a ternary complex containing otefin, BAF and lamin, licensing the recruitment of otefin to reassembling nuclei during mitotic exit. Failure in this event delays NER and leads to early embryonic lethality. The mechanism identified could constitute an important means of regulation of post-mitotic nuclear reassembly conserved across species.

## 2. Results

### 2.1. Identification of PP2A-Tws interactors

To guide the discovery of novel PP2A-Tws targets, we sought to identify specific interactors. Because we were interested in functions of PP2A-Tws in mitosis, we chose to do our purifications from early *Drosophila* embryos, which spend much of their time in mitosis. At this stage, 13 rounds of mitosis occur synchronously in a syncytium, at intervals of approximately 10 min. We used transgenic lines that expressed Tws-GFP and GFP-Tws under the control of the upstream activating sequence (UASp). These fusion proteins were previously shown to be functional as their transgenic expression induced by a ubiquitous driver rescued the development of *tws* mutant flies [55]. Expression was induced using the maternal driver *matα4-GAL-VP16*, which allowed production of the proteins during oogenesis and early embryogenesis. Embryos aged 0 to 2 h were collected and submitted to GFP affinity purification. Purified products were analysed by mass spectrometry. Embryos expressing GFP alone were used as a control. The experiment was done three times, allowing for statistical analysis (electronic supplementary material, table S1).

This analysis confirmed that Tws forms a complex with the core PP2A subunits: the structural PP2A-29B and the catalytic Mts (figure 1*a–d*). Several previously demonstrated or proposed mitotic substrates of *Drosophila* PP2A-Tws were also identified as specific interactors of Tws-GFP (figure 1*d* left, green names). These include the nuclear envelope-associated proteins BAF, Lamin and the nuclear pore complex protein Nup107 [43]. These results are consistent with the notion that PP2A-Tws promotes NER after mitosis [8]. The calmodulin-dependent kinase CaMKII and the cohesin Vtd/Scc1 are other known targets of PP2A-Tws that were identified [56,57]. Among specific interactors of Tws-GFP, we also recognized orthologues of several known substrates of PP2A-B55 enzymes in humans (electronic supplementary material, figure S1, red names) [10,14,58]. In comparison, few known targets of PP2A-Tws/B55 were identified as interaction partners of GFP-Tws (figure 1*d* right). Thus, the N-terminal GFP tag may hinder the stable interaction of substrates at the Tws-binding pocket. The large number of interactors of Tws-GFP identified is consistent with the numerous functions of PP2A-Tws/B55 in various cellular processes [10]. However, a large proportion of these interactions is probably indirect. For this reason, it was difficult to identify novel mitotic targets of PP2A-Tws with high confidence based on these data alone.

### 2.2. Identification of PP2A-Tws-dependent phosphoproteins

To identify potential substrates of PP2A-Tws, we used a proteomic approach with *Drosophila* cells in culture (figure 2*b*). D-Mel cells were transfected with dsRNA against Tws or with non-target dsRNA as control. After 4 days, cells were harvested and lysed, and proteins were digested with trypsin. Phosphopeptides were enriched on a TiO_2_ resin and analysed by mass spectrometry. We initially attempted to use a stable isotope labelling in cells in culture (SILAC) approach for direct quantitative measurements comparing two samples [59]. However, the synthetic medium required for SILAC dramatically increased the doubling time of the cells (our unpublished observations). Instead, we used a label-free analysis strategy, where quadruplicates for each condition allowed for the generation of quantitative data (electronic supplementary material, table S2).

In this analysis, phosphopeptides containing PP2A-Tws dephosphorylation sites are expected to be more abundant in the Tws RNAi samples relative to controls (figure 2*c*, top-right corner of the volcano plot). Interestingly, sites found to be hyperphosphorylated after Tws depletion had a strong tendency to immediately precede a proline residue, consistent with the reported ability of PP2A-B55 enzymes to dephosphorylate CDK1 sites (electronic supplementary material, figure S2*a*). However, hyperphosphorylated sites identified after Tws RNAi do not necessarily reflect a role of PP2A-Tws in mitotic exit because this phosphatase likely functions in the regulation of various cellular processes, even during interphase. To help the identification of PP2A-Tws targets in mitotic exit, we included in our phosphoproteomic analysis, the RNAi knockdown of Gwl and Endos. Both proteins function together to inactivate PP2A-Tws during mitotic entry, and genetic analysis indicated that this is their only essential function in mitosis (figure 2*a*) [60]. In this way, phosphopeptides containing PP2A-Tws dephosphorylation sites are expected to be less abundant in the Gwl RNAi and Endos RNAi samples relative to controls (figure 2*c*, top-left corner of the volcano plots). Thus, in the clustering of the data, we were particularly interested in phosphopeptides enriched after Tws RNAi and depleted after Gwl and/or Endos (figure 2*d*, purple branches). Of the 42 phosphopeptides identified and grouped in this cluster, 14 belonged to 12 proteins also identified as significantly enriched interactors of Tws-GFP in our purifications: otefin (2 peptides), Lost (2 peptides), Nup358, Klc, HBS1, chb (Orbit), Akap200, Ctf4, MEP-1, Map205, CG7414 (eIF2A) and Nocte (figure 1*c,d*, blue names). These proteins constitute likely substrates of PP2A-Tws. As expected, Endos, a known PP2A-Tws substrate, was also detected as hyperphosphorylated at the expected site after Tws depletion and hypophosphorylated after Gwl depletion (figure 2*d*, in blue).

As an orthogonal approach, we used chemical inhibition. We treated cells for 1 h with okadaic acid (OA), which inhibits all forms of PP2A and also other PPP family phosphatases [61]. Phosphoproteomic analysis identified hundreds of sites that became hyperphosphorylated following OA treatment compared with DMSO control (figure 2*e*). Many of the Tws-dependent sites were also OA-dependent, including those identified in otefin, which will be characterized below (electronic supplementary material, figure S2*b*). We consider that sites identified in both analyses are likely to be dephosphorylated by PP2A-Tws.

### 2.3. Otefin phosphorylation near its LEM domain is regulated by PP2A-Tws

**Figure 3.**
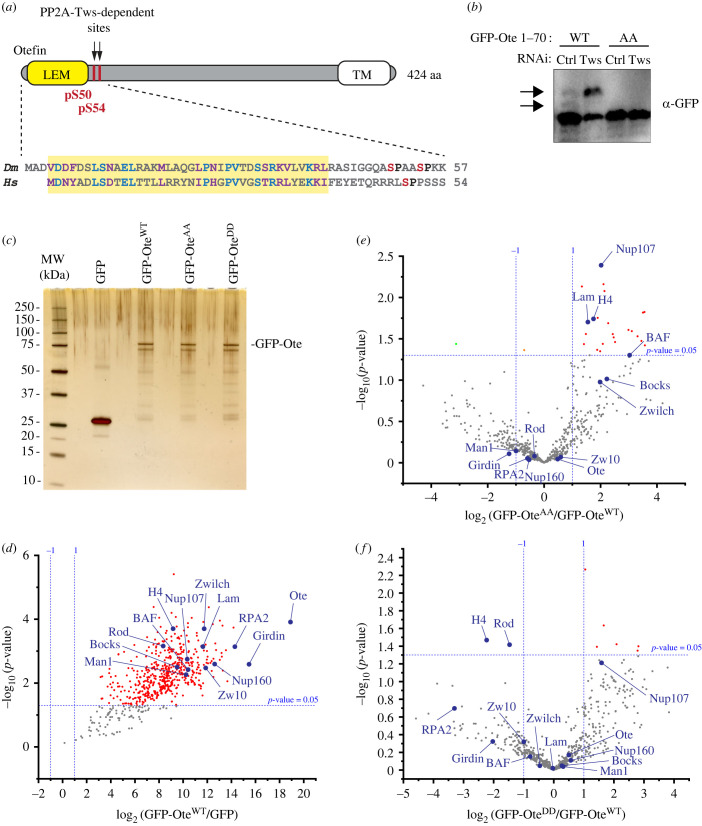
PP2A-Tws-dependent phosphosites on otefin regulate its protein interaction profile. (*a*) PP2A-Tws-dependent pSer50 and pSer54 (red) are located immediately C-terminal to the LEM domain in otefin. A sequence alignment with human Emerin shows that a known phosphorylation site (Ser49, red) is similarly located. TM: transmembrane domain. (*b*) Otefin phosphorylation at Ser5-Ser54 is sensitive to PP2A-Tws activity. D-Mel cells were transfected as indicated and their lysates were submitted to SDS-PAGE in the presence of Phostag followed by an anti-GFP Western blot. Arrows: presumed phosphorylated forms. (*c*) Purification of GFP-Ote variants from *Drosophila* embryos. Embryos aged 0 to 2 h expressing GFP or GFP-Ote WT, AA or DD were submitted to GFP affinity purifications. The gel shows 5% of the total purification products on a silver-stained 4–12% acrylamide SDS-PAGE gel. The rest of the samples were submitted to mass spectrometric analyses. (*d*) Identification of proteins significantly enriched with GFP-Ote compared to GFP alone. Data points are from three independent experiments. (*e*,*f*) Identification of proteins significantly enriched or depleted with GFP-Ote^AA^ versus GFP-Ote^WT^ (*e*) or with GFP-Ote^DD^ versus GFP-Ote^WT^ (*f*). Data points are from three independent experiments. In (*d*–*f*), proteins of interest discussed in the text are labelled.

The intersection of our interaction and phosphorylation analyses pointed to otefin as a likely target of PP2A-Tws. Otefin is a component of the inner nuclear membrane, and we have previously shown that PP2A-Tws promotes the reassembly of the nuclear envelope [43]. We therefore became interested in the regulation of otefin by PP2A-Tws. Both PP2A-Tws-dependent phosphopeptides identified were phosphorylated at Ser50, and one of them was also phosphorylated at Ser54. Ser50 and Ser54 are located immediately after the LEM domain of otefin in its primary structure (figure 3*a*). Both sites lie in a consensus motifs for phosphorylation by CDKs (minimal: S/T-P or optimal: S/T-P-X-K/R) [1]. Ser54 of otefin was previously shown to be phosphorylated by Cyclin B-CDK1 [62]. Moreover, sequence alignment with human emerin reveals one site, Ser49, which is similarly located after the LEM domain and lies in a minimal CDK motif (figure 3*a*). The PhosphoSitePlus database indicates that human emerin was frequently reported to be phosphorylated at Ser49 [63]. For these reasons, we hypothesized that phosphorylation at otefin at Ser50–Ser54 could serve an important biological function.

To visualize phosphorylation at these sites and its dependence on PP2A-Tws, we used SDS-PAGE with the addition of Phostag. We expressed in D-Mel cells a small N-terminal fragment of otefin in fusion with GFP (GFP-Ote 1–70). We observed a faint band at a higher molecular weight for GFP-Ote 1–70 that was lost upon mutation of Ser50 and Ser54 (figure 3*b*, arrow). Moreover, RNAi depletion of Tws induced a marked increase in the intensity of this higher band for GFP-Ote^WT^ 1–70, but not for GFP-Ote^AA^ 1–70. We conclude that otefin dephosphorylation at Ser50 and/or Ser54 is strongly dependent on PP2A-Tws.

### 2.4. Identification of phosphorylation-dependent interactors of otefin

To explore how phosphorylation of otefin (Ote) at the identified sites regulates its protein interactions, we generated transgenic fly lines expressing GFP-Ote, either WT (GFP-Ote^WT^) or with Ser50–Ser54 mutated into non-phosphorylatable alanine residues (GFP-Ote^AA^) or phosphomimetic aspartic acid residues (GFP-Ote^DD^). We collected embryos from these flies and used them in GFP affinity purifications followed by mass spectrometry analysis as described above for Tws (figure 3*c*). We identified several proteins significantly enriched in the GFP-Ote^WT^ purification products relative to GFP alone (figure 3*d*; electronic supplementary material, figure S3 and table S3). BAF and lamin, two proposed direct interactors of otefin, were identified as expected [49,64]. Other chromatin-associated proteins were also identified. Although some of them could potentially interact with otefin directly, they may also be co-purified because BAF and Lamin bridge interactions with chromatin proteins. For example, it is likely the case for Histone H4, as BAF was shown to bind histones in human cells [65,66]. We also found other NE proteins in complex with otefin, including its two LEM-domain protein orthologues Bocksbeutel (Bocks) and MAN1, and nucleoporins of the Nup107-Nup160 complex. Interestingly, our proteomic analysis identified a clear and novel association between otefin and girdin, an actin-binding protein conserved in humans [67,68]; girdin was the most enriched protein in the GFP-otefin purification products (figure 3*d*). Finally, we note our identification of all three members of the RZZ complex (Rod-Zw10-Zwlich), known to function in the attachment of kinetochore to microtubules [69]. Several other proteins that enriched with otefin were abundant RNA metabolism proteins and metabolic enzymes for which we have no functional hypotheses.

To identify otefin interactors that depend on the phosphorylation status of Ser50 and Ser54, we compared the abundance of proteins specifically co-purified with GFP-Ote^AA^ versus GFP-Ote^WT^ or GFP-Ote^DD^ versus GFP-Ote^WT^. We found that higher levels of BAF and Lamin were co-purified with GFP-Ote^AA^ compared with GFP-Ote^WT^ (figure 3*e*). By contrast, most other putative interactors of otefin were not significantly affected in their co-purified levels between GFP-Ote^AA^ and GFP-Ote^WT^. Conversely, BAF levels tended to be depleted in the GFP-Ote^DD^ purification product compared to GFP-Ote^WT^, although the difference was not statistically significant (figure 3*f*). Histone H4, a known BAF interactor, was also significantly enriched with GFP-Ote^AA^ and depleted with GFP-Ote^DD^, compared with GFP-Ote^WT^.

### 2.5. PP2A-Tws-dependent phosphosites on otefin regulate its interaction with barrier-to-autointegration factor

**Figure 4.**
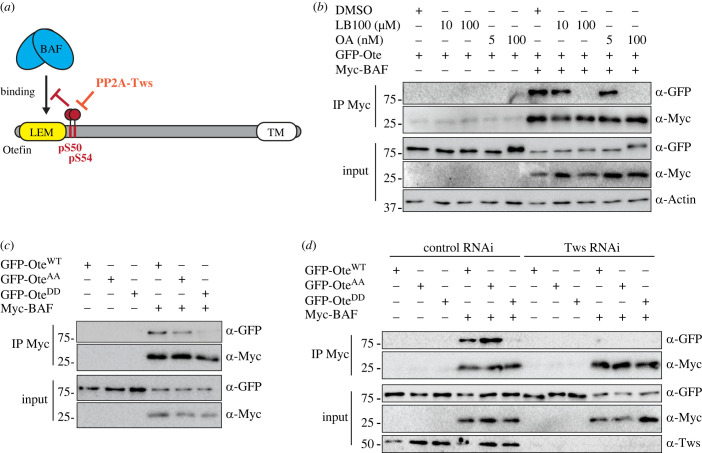
PP2A-Tws-dependent phosphosites on otefin regulate its interaction with BAF. (*a*) Hypothetical model where phosphorylation of otefin at Ser50–Ser54 inhibits its interaction with BAF, and where dephosphorylation by PP2A-Tws would promote the interaction. (*b*) Phosphatase inhibition disrupts the otefin–BAF interaction. D-Mel cells were transfected to express the indicated proteins and were treated with chemical inhibitors as indicated. Cells were then submitted to anti-Myc immunoprecipitation and products were analysed by Western blotting. (*c*) Phosphomimetic mutations in GFP-otefin abrogate its interaction with Myc-BAF. Cells were transfected to express the indicated proteins and submitted to anti-Myc immunoprecipitation. Products were analysed by Western blotting. (*d*) Depletion of Tws abrogates the interaction between otefin and BAF. Cells were transfected as indicated and submitted to anti-Myc immunoprecipitation. Products were analysed by Western blotting.

The above results suggested that reversible phosphorylation of otefin at Ser50–Ser54 regulates its interaction with BAF. Moreover, these phosphorylation sites, within CDK motifs, are immediately adjacent to otefin's LEM domain, which is known to interact with BAF in all LEM domain proteins examined [44,70]. Thus, we hypothesized that phosphorylation at Ser50 and Ser54 may disrupt the interaction of otefin with BAF during mitotic entry. Conversely, dephosphorylation of these sites by PP2A-Tws would promote the otefin–BAF interaction during mitotic exit, as the nuclear envelope reassembles (figure 4*a*).

To begin testing this idea, we used a co-immunoprecipitation assay in D-Mel cells transfected with Myc-BAF and GFP-otefin. As expected, GFP-Ote could be co-purified with Myc-BAF. To test if PP2A activity was required for the otefin–BAF interaction, we treated cells with OA (a PPP inhibitor with broad specificity) or with LB100, which inhibits more selectively PP2A and PP5 [71,72]. We found that both inhibitors strongly abrogated the otefin–BAF interaction, consistent with the idea that PP2A activity promotes the interaction (figure 4*b*). Interestingly, OA (100 nM) induced an upshift of the GFP-Ote band, suggesting its hyperphosphorylation.

To test if phosphorylation of otefin at Ser50 and Ser54 negatively regulates its interaction with BAF, we used the non-phosphorylatable (Ote^AA^) and phosphomimetic (Ote^DD^) mutants. We found that Myc-BAF co-purified less GFP-Ote^DD^ than GFP-Ote^WT^ or GFP-Ote^AA^ (figure 4*c*). These results suggest that phosphorylation of otefin at Ser50 and Ser54 negatively regulates its interaction with BAF.

OA and LB100 are thought to inhibit all forms of PP2A, indiscriminately of its associated regulatory subunit. To test if the otefin–BAF interaction is regulated by PP2A-Tws, we conducted the same assay as above following RNAi silencing of Tws. We found that the otefin–BAF interaction was abrogated by the loss of Tws (figure 4*d*). This is consistent with the idea that dephosphorylation of otefin by PP2A-Tws promotes the otefin–BAF interaction. However, Tws depletion abolished the otefin–BAF interaction even when the otefin phosphorylation sites were mutated to alanine residues. This suggests that there are other PP2A-Tws-dependent phosphorylation sites in otefin or in BAF that may be involved in regulating the interaction. Phosphorylation of BAF in its N-terminus may be responsible for this effect as it is known to negatively regulate its interactions with LEM-D proteins, and its recruitment to the reassembling nuclear envelope in human cells [44,46]. Moreover, we previously reported that PP2A-Tws promotes BAF recruitment to reassembling nuclei in *Drosophila* cells [43]. Thus, PP2A-Tws may promote the otefin–BAF interaction by redundant mechanisms.

### 2.6. PP2A-Tws-dependent phosphosites on otefin regulate the formation of a complex containing barrier-to-autointegration factor, lamin and additional otefin

**Figure 5.**
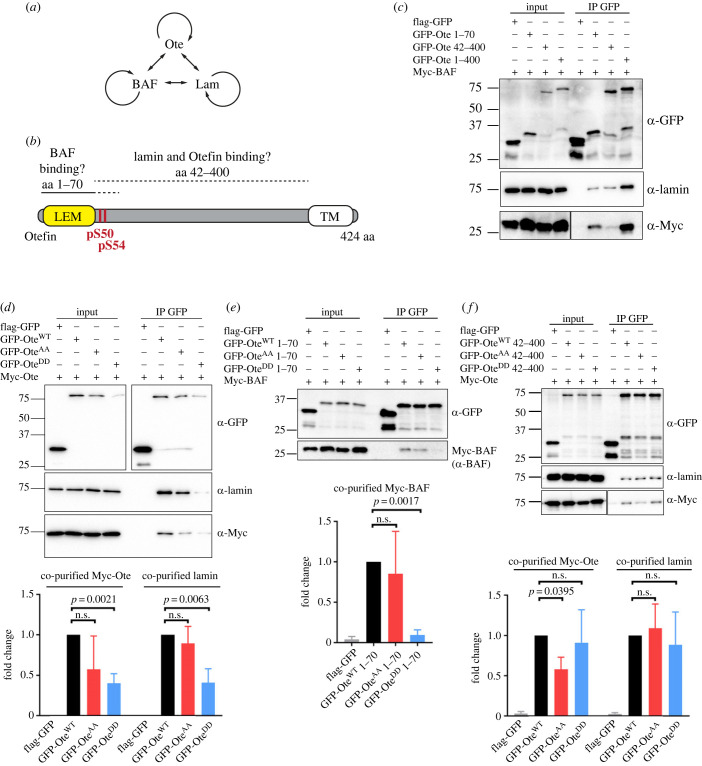
PP2A-Tws-dependent phosphosites on otefin regulate the formation of a complex containing BAF, lamin and additional otefin. (*a*) Otefin, BAF and lamin are thought to interact with each other and to engage in homomeric interactions. (*b*) Truncations 1–70 and 42–400, thought to bind BAF and lamin + otefin, respectively, were designed to help dissect the direct impact of phosphorylation at Ser50–Ser54. (*c*–*f*) Cells were transfected as indicated and submitted to GFP affinity purifications. Purification products were analysed by Western blotting. Quantifications shown correspond to ratios of the co-purified protein relative to the amounts of GFP-tagged protein, where values obtained with GFP-Ote^WT^ variants are normalized to one. Error bars: standard deviation. *p*-values are from paired *t*-tests. n.s.: non-significant (*p* > 0.05). (*c*) The association of BAF and Lamin is stronger with otefin 1–400 compared with otefin 1–70 or otefin 42–400. Note that lower MW bands below GFP-fused proteins likely correspond to breakdown products containing anti-GFP epitopes. (*d*) The interactions of lamin and otefin with otefin are abrogated by phosphomimetic mutations of Ser50–Ser54. Quantifications shown are averages of 4 experiments. (*e*) The interactions of BAF with otefin 1–70 is abrogated by phosphomimetic mutations of Ser50–Ser54. Quantifications shown are averages of three experiments. (*f*) The interactions of lamin and otefin with otefin 42–400 are not abrogated by phosphomimetic mutations of Ser50–Ser54.

In addition to interacting with BAF and other proteins, human emerin also interacts with itself, forming homomers at the nuclear lamina [73–75]. Thus, we considered that otefin may also engage in homomeric interactions, in addition to binding BAF and lamin. Since BAF forms dimers and lamin forms polymers, large complexes containing multiple copies of otefin, BAF and lamins could assemble in a cooperative manner and complicate the interpretation of co-purification results (figure 5*a*). To better understand the impact of Ser50–Ser54 phosphorylation on Otefin interactions, we used a truncation analysis. We generated constructs for the expression of GFP-Ote 1–70, GFP-Ote 42–400 and GFP-Ote 1–400, all of which containing Ser50 and Ser54. The 1–70 region contains the region homologous to the LEM domain of human emerin shown to bind BAF [73,76]. The 42–400 region is homologous to the region shown to be sufficient to bind lamins and emerin [73] (figure 5*b*). Using these fusion proteins, we proceeded to co-purification experiments on transfected cells. We found that all three truncations of GFP-Ote specifically co-purified Myc-BAF, Myc-Ote and Lamin. However, GFP-Ote 1–400 co-purified all three proteins more efficiently than GFP-Ote 1–70 or GFP-Ote 42–400 (figure 5*c*; electronic supplementary material, figure S4). This result is consistent with cooperative binding in the formation of a complex containing otefin, BAF and lamin (figure 5*a*). Nevertheless, Myc-BAF co-purified more strongly with GFP-Ote 1–70 compared with GFP-Ote 42–400, while Myc-Otefin and lamin co-purified more strongly with GFP-Ote 42–400 compared with GFP-Ote 1–70, as expected (figure 5*c*; electronic supplementary material, figure S4).

Also consistent with the cooperative binding model, we found that the phosphomimetic mutations in GFP-Ote abrogated its interaction with Myc-Ote and with lamin, in addition to Myc-BAF (figures 4*c* and 5*d*). To identify which interactions of otefin are directly dependent on its phosphorylation at Ser50–Ser54, we used variants of GFP-Ote 1–70 and GFP-Ote 42–400, which preferentially interact with BAF or with otefin and lamin, respectively. We found that the S50D-S54D phosphomimetic mutations in GFP-Ote 1–70 decreased its ability to interact with Myc-BAF (figure 5*e*). By contrasts, the phosphomimetic mutations in GFP-Ote 42–400 did not decrease its ability to interact with Myc-Ote or Lamin (figure 5*f*). These results suggest that phosphorylation of Otefin at Ser50–Ser54 negatively regulates its interaction with BAF, thereby also indirectly affecting formation of the otefin–BAF–lamin complex.

### 2.7. PP2A-Tws regulates the recruitment of otefin and barrier-to-autointegration factor to reassembling nuclei after mitosis

**Figure 6.**
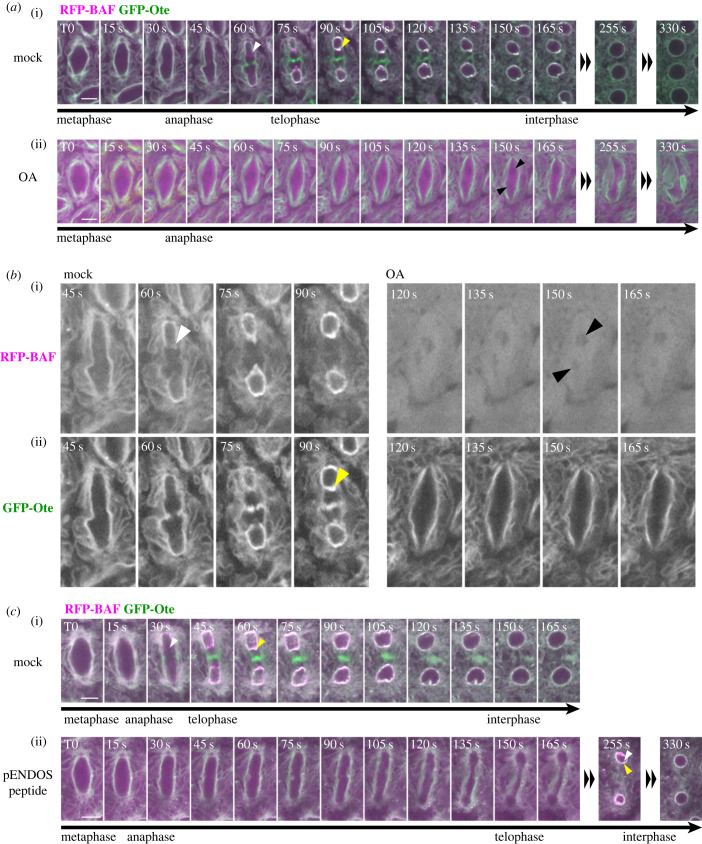
PP2A-Tws regulates the recruitment of otefin and BAF to reassembling nuclei after mitosis in embryos. (*a*) Time-lapse images from an embryo expressing GFP-Ote and RFP-BAF and microinjected with a mock solution of 15% DMSO in PBS ((i), electronic supplementary material, video S2) or with a solution of 100 µM OA in 15% DMSO in PBS ((ii), electronic supplementary material, video S4). After mock injection, RFP-BAF is recruited first to the core region (white arrowhead) and GFP-Ote is recruited later (yellow arrowhead). After OA injection, RFP-BAF and GFP-Ote fail to be recruited around segregated chromosomes (black arrowheads). (*b*) Enlarged images selected from (*a*), with separated GFP and RFP channels. (*c*) Time-lapse images from an embryo expressing GFP-Ote and RFP-BAF and microinjected with a mock solution of PBS ((i), electronic supplementary material, video S3) or with a phospho-Endos (pENDOS) peptide capable of inhibiting PP2A-Tws (10 mM in PBS, (ii), electronic supplementary material, video S5). Note that the recruitment of RFP-BAF (white arrowhead) and GFP-Ote (yellow arrowhead) to reassembling nuclei is delayed in the presence of the pENDOS peptide. Scale bars: 5 µm.

To monitor the dynamics of otefin during mitosis, we used live imaging. We filmed mitosis in embryos expressing GFP-Ote along with RFP-BAF (figure 6*a*, mock series; electronic supplementary material, videos S1–S3). As expected, GFP-Ote was enriched at the nuclear envelope and at intracellular membranes (putative ER) in interphase, consistent with its previously reported localization [77]. During mitotic entry and until anaphase onset, GFP-Ote remained localized to the spindle envelope, which corresponds to the nuclear envelope that is fenestrated at poles to allow the spindle to connect chromosomes and centrosomes [77,78]. RFP-BAF showed a similar localization, but a larger fraction of this protein appeared to be cytoplasmic during early mitosis. During late anaphase, RFP-BAF was rapidly recruited to segregated chromosomes (figure 6*a*,*b*, mock series, white arrowheads). At that stage, GFP-Ote was not yet recruited to the region of reassembling nuclei that is proximal to the spindle (the inner core region). Within 1 min, GFP-Ote rejoined RFP-BAF at the inner core region as two separate nuclear envelopes reassembled from the spindle envelope (figure 6*a*,*b*, mock series, yellow arrowheads).

To begin to test whether PP2A phosphatase activity was required for this localization dynamics, we injected OA in these syncytial embryos at the metaphase stage. We could discern nuclei that underwent anaphase from fluorescence exclusion zones produced by segregating, condensed chromosomes (figure 6*a*,*b*, black arrowheads, electronic supplementary material, video S4). However, RFP-BAF failed to be recruited to segregated chromosomes, remaining completely dispersed, and GFP-Ote never reached the inner core region as the nuclear envelope failed to reassemble. These results are consistent with the role of PP2A in the dephosphorylation of BAF and its recruitment to reassembling nuclei [43,48].

However, OA also inhibits other phosphatases and no small molecule inhibitor exists that would be selective to PP2A-B55/Tws. To achieve selective inhibition of PP2A-Tws, we injected a peptide derived from Endos and phosphorylated at its Gwl site (pEndos: aa 41–80, pSer67). We observed delays in the recruitment of RFP-BAF and GFP-Ote at reassembling nuclei after anaphase (figure 6*c*, bottom (255 s); electronic supplementary material, video S5). Nuclei eventually reassembled and both proteins were recruited, suggesting either that PP2A-Tws eventually dephosphorylated enough pEndos to escape inhibition, or that another phosphatase can compensate for the loss of PP2A-Tws activity. Altogether, our results confirm that PP2A-Tws promotes NER by targeting both BAF and Otefin.

### 2.8. PP2A-Tws-dependent sites in otefin regulate its recruitment to reassembling nuclei after mitosis

**Figure 7.**
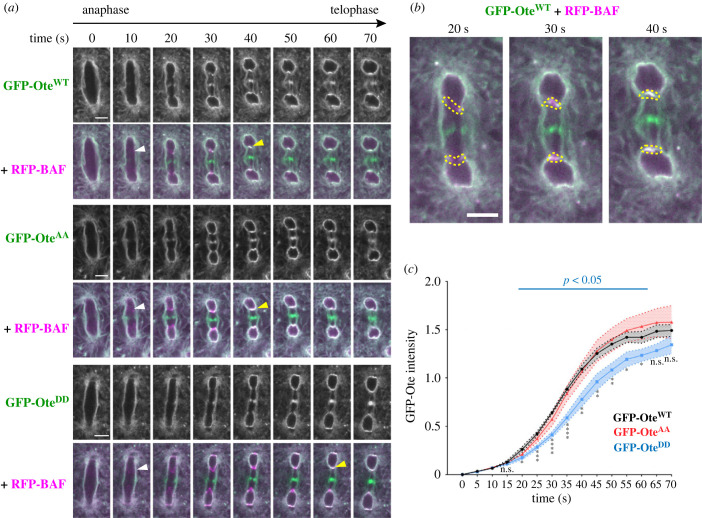
PP2A-Tws-dependent phosphosites on otefin regulate its recruitment to reassembling nuclei after mitosis in embryos. (*a*) Time-lapse images from embryos expressing GFP-Ote WT, AA and DD. *T*_0_ was set as the time immediately preceding the first time when RFP-BAF becomes visible at the inner core region (white arrowheads). GFP-Ote recruitment progresses from the sides inwards until it reaches the centre of the core regions (yellow arrowheads). (*b*) Illustration of the quantification method using enlarged images from (*a*). Inner core regions were drawn from side to side of the spindle envelope around the area corresponding to RFP-BAF localization. For each mitotic figure quantified, fluorescence at both inner core regions were measured and averaged for each time point. (*c*) Quantification results of GFP-Ote fluorescence at the inner core region from films such as shown in (*a*). Ten representative nuclei from five or six different embryos were quantified for each genotype. Error areas: S.E.M. Scale bars: 5 µm. * *p* < 0.05, ** *p* < 0.01, *** *p* < 0.001, **** *p* < 0.0001 from Welch's *t*-tests. n.s.: non-significant (*p* > 0.05).

To test if regulation at Ser50–Ser54 impacts the dynamics of otefin during mitosis, we filmed mitosis in embryos expressing GFP-Ote WT, AA or DD, along with RFP-BAF (figure 7*a*). We compared the fluorescence of GFP-Ote^DD^ versus GFP-Ote^WT^ at the inner core region, taking the onset of RFP-BAF recruitment as a reference time (figure 7*b*). We found that the recruitment of GFP-Ote^DD^ was significantly delayed compared to GFP-Ote^WT^ (figure 7*c*). By contrast, the dynamics of GFP-Ote^AA^ was similar to that of GFP-Ote^WT^. The timing of RFP-BAF recruitment relative to anaphase did not appear to be altered by the expression of the GFP-Ote variants. These results suggest that dephosphorylation of otefin facilitates its recruitment to reassembling nuclei by promoting its interaction with BAF. Moreover, because GFP-Ote is a transmembrane protein, it provides a marker for the nuclear envelope, and therefore, our results suggest that PP2A-Tws-dependent dephosphorylation of otefin at Ser50–Ser54 determines the timing of NER, at least at the inner core region. However, in this experiment, the delay observed in the recruitment of GFP-Ote^DD^ may not reflect the full delay that would result from a failure to dephosphorylate the entire pool of otefin because GFP-Ote^DD^ is able to interact with endogenous Ote.

**Figure 8.**
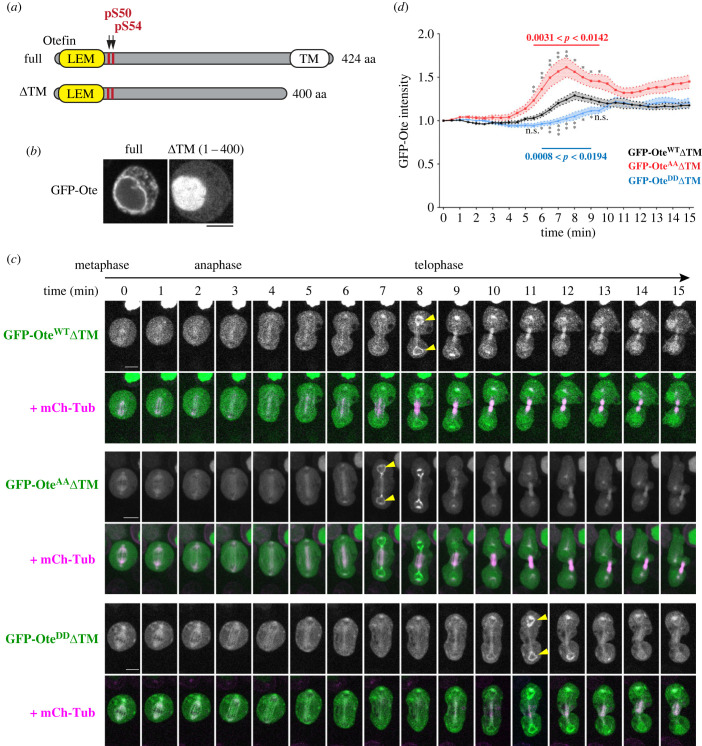
PP2A-Tws-dependent phosphosites on otefin regulate its recruitment to reassembling nuclei independently from its transmembrane domain. (*a*) Truncation of the putative transmembrane domain in otefin (ΔTM). (*b*) While GFP-Ote full length localizes to the NE in interphase, GFP-OteΔTM is dispersed in the nucleus. (*c*) Time-lapse images of cells expressing GFP-OteΔTM WT (electronic supplementary material, video S6), AA or DD and mCherry-tubulin and undergoing mitosis. *T*_0_ was set as the time point just prior to spindle elongation. Yellow arrowheads: maximum intensity of GFP fluorescence at reassembling nuclei. Scale bars: 5 µm. (*d*) Quantification of the GFP fluorescence at reassembling nuclei from cells as shown in (*c*). Thirty cells were quantified for each cell line. Error areas: s.e.m. Scale bars: 5 µm. * *p* < 0.05, ** *p* < 0.01, *** *p* < 0.001 from Welch's *t*-tests. n.s.: non-significant (*p* > 0.05).

To visualize the effects of the phosphorylation sites in otefin on its recruitment via protein interactions but independently from its membrane localization, we used the GFP fusion of otefin deleted of its transmembrane domain (GFP-Ote 1–400 = GFP-OteΔTM) (figure 8*a*). While GFP-Ote^WT^ localized to the nuclear envelope in D-Mel cells, GFP-OteΔTM did not, but was instead enriched in the nucleoplasm in interphase (figure 8*b*), consistent with a previous report [79]. After mitotic NEBD, GFP-OteΔTM was dispersed throughout the cell. In telophase, GFP-OteΔTM was transiently recruited to segregated chromosomes (figure 8*c*; electronic supplementary material, video S6). We presumed that this localization of GFP-OteΔTM must reflect its formation of a protein complex containing BAF, which is known to be recruited with a similar timing [43]. Consistent with this notion, we found that GFP-Ote 1–70 (which contains the LEM domain and interacts with BAF) was recruited to chromosomes in telophase, while GFP-Ote 42–400 (which lacks the LEM domain and cannot interact with BAF directly), was not recruited (electronic supplementary material, figure S5). Therefore, the recruitment of otefin to reassembling nuclei depends on its LEM domain. Interestingly, GFP-Ote 1–400 (ΔTM) was recruited more strongly than GFP-Ote 1–70, consistent with cooperativity in complex formation between otefin, BAF and lamin (electronic supplementary material, figure S5; figure 5). As expected, we found that the recruitment of GFP-Ote^DD^ΔTM was delayed compared to GFP-Ote^WT^ΔTM (figure 8*c*,*d*). The observed recruitment of GFP-Ote^DD^ΔTM likely reflects its residual ability to interact with endogenous otefin and lamin, Conversely, the recruitment of GFP-Ote^AA^ΔTM was advanced and appeared on average stronger compared to GFP-Ote^WT^ΔTM (figure 8*d*). These results indicate that dephosphorylation of otefin by PP2A-Tws at Ser50–Ser54 promotes otefin recruitment to reassembling nuclei by allowing otefin to form a complex with BAF.

### 2.9. PP2A-Tws-dependent phosphosites on otefin regulate its essential functions *in vivo*

**Figure 9.**
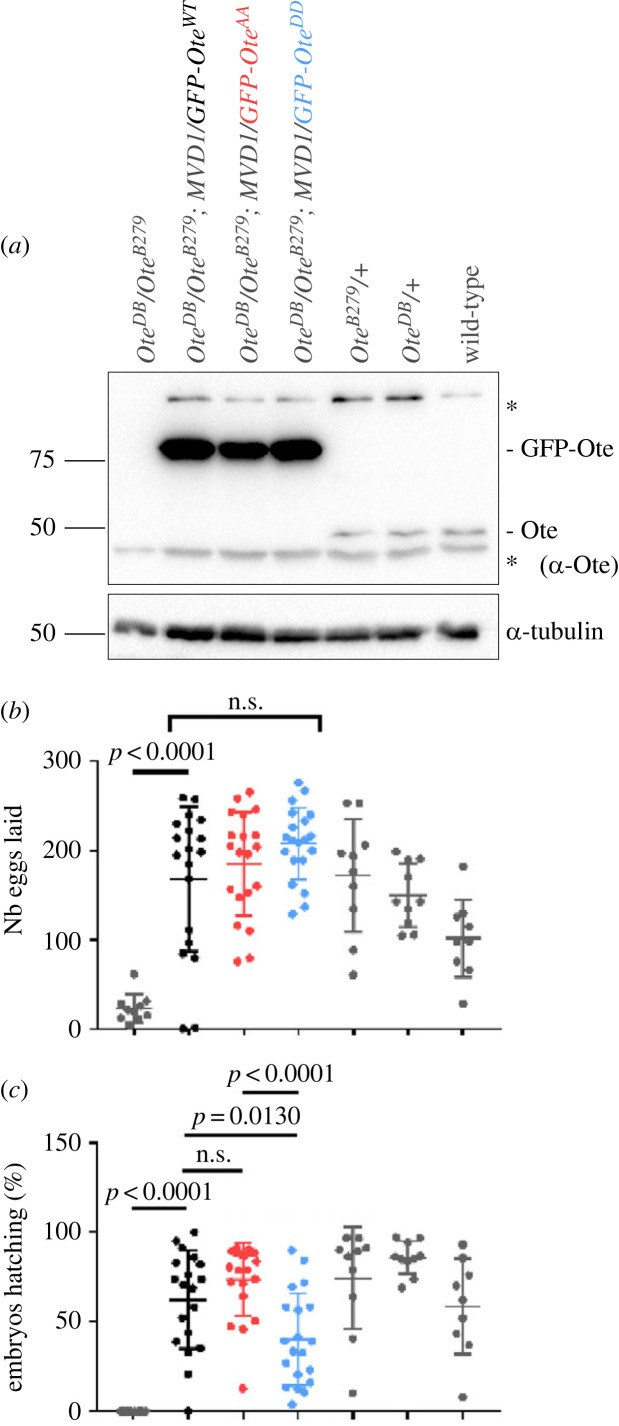
PP2A-Tws-dependent phosphosites on otefin regulate its essential functions *in vivo.* (*a*) Western blot showing otefin expression in ovaries from females of the indicated genotypes. The expression of the *UASp-GFP-Ote* transgenes was induced by the MVD1 maternal driver. The positions of GFP-otefin and endogenous otefin are indicated. * non-specific bands. (*b*) Numbers of eggs laid by females of the indicated genotypes. Data points indicate the number or eggs laid over 3 days by individual females. Bars: averages ± s.d. (*c*) Numbers of hatched embryos from females of the indicated genotypes. Data points indicate the percentage of embryos from individual females that hatched over 3 days. Bars: averages ± s.d. Indicated *p*-values were obtained from unpaired *t*-tests. n.s.: non-significant (*p* > 0.05).

We sought to test the importance of otefin phosphoregulation for its functions *in vivo*. Otefin is not essential for viability because its absence is compensated by the presence of its two orthologues Bocks and MAN1, both LEM domain proteins of the inner nuclear membrane [80]. However, *ote* mutant flies are female-sterile [81]. *ote^B279^/ote^DB^* females produce a reduced number of eggs, none of which hatch. We expressed GFP-Ote in the female germline using the *GAL4::VP16-nanos.UTR* (MVD1) driver, which allows expression throughout female germline development, oogenesis and early embryogenesis [82] (figure 9*a*). We found that expression of GFP-Ote^WT^ completely rescued egg production by *ote^B279^/ote^DB^* mutant females, indicating that N-terminal fusion of GFP preserves otefin function (figure 9*b*). GFP-Ote^AA^ and GFP-Ote^DD^ also fully rescued egg production, indicating that phosphoregulation of otefin at Ser50–Ser54 is not required for oogenesis. However, a smaller fraction of embryos laid by *ote* mutant females expressing GFP-Ote^DD^ hatched, compared with embryos laid by *ote* mutant females expressing GFP-Ote^WT^ or GFP-Ote^AA^ (figure 9*c*). These results indicate that dephosphorylation of otefin at Ser50–Ser54 by PP2A-Tws is important, although not absolutely essential, in the context of early embryonic development which relies on rapid mitotic cycles.

## 3. Discussion

In this work, we used a proteomic strategy to identify potential substrates of PP2A-Tws. Tws interactors and Tws-dependent phosphorylation sites were uncovered. Both approaches identified otefin. We found that phosphorylation of otefin at sites immediately C-terminal to its LEM domain negatively regulates its ability to interact with BAF and to form a complex with lamin and additional otefin. We validated that dephosphorylation of these sites depends on PP2A-Tws. Microscopy and genetic experiments revealed that dephosphorylation of otefin is required for its timely recruitment to reassembling nuclei and for embryonic development. To our knowledge, this may be the first documented case of any protein dephosphorylation event shown to be required for the development of an animal.

Previous work has shown that B55 exposes a binding surface that is used by PP2A-B55 to dock onto its dephosphorylation substrates [13,83]. Consistent with this notion, we could capture PP2A-Tws interactions with several of its targets by affinity purifications. It was reported that PP2A-B55/Tws efficiently dephosphorylates CDK sites [15–20]. Consistent with this idea, almost all Tws-dependent sites identified were threonine and serine residues followed by a proline residue. However, while PP2A-B55 was shown to prefer phospho-threonines to phospho-serines [20,21], most PP2A-Tws sites we identified are phospho-serines. This may be merely because phosphorylation occurs more frequently at serines than at threonines in the *Drosophila* proteome. Indeed, our analyses detected around 80% of phospho-serines versus less than 20% of phospho-threonines, consistent with previous results in *Drosophila* or in human cells [84–89].

Our analysis suggests other mitotic targets of PP2A-Tws in addition to otefin. Candidate targets that were identified as PP2A-Tws interactors and as phosphoproteins enriched upon Tws inactivation but depleted upon Gwl and/or Endos inactivation should be viewed with the highest confidence. The microtubule-associated protein Map205 is one of them. The PP2A-Tws-dependent site we identified on Map205 (Ser283, a CDK motif) was previously shown to regulate an interaction with Polo kinase. Phosphorylation of this site in mitosis frees Polo from an interaction with Map205 which inhibits Polo and sequesters it on microtubules. This release facilitated Polo's essential functions in mitosis and cytokinesis [90,91]. Thus, dephosphorylation of Map205 by PP2A-Tws may promote the binding of Polo to Map205 during mitotic exit, where Polo returns to its inactive state. Another candidate mitotic target of PP2A-Tws we found is chb/Orbit, a motor protein that regulates spindle microtubules [92]. We also identified Ctf4, an essential DNA replication factor, which may be a target of PP2A-Tws in its emerging role in the regulation of S-phase [10,93].

The two PP2A-Tws-dependent sites we identified in otefin match the consensus motif for phosphorylation by CDKs. Moreover, one of them, Ser54, was shown to be phosphorylated by CycB-CDK1 [62]. CycB-CDK1 is well known for its pivotal role in mitotic entry and NEBD [1]. Conversely, PP2A-Tws is inactivated by Gwl and Endos during mitotic entry and becomes reactivated during mitotic exit [94]. Thus, we propose that phosphorylation of otefin by CycB-CDK1 during mitotic entry contributes to disrupt its interactions with BAF and lamin as part of NEBD during mitotic entry, while the dephosphorylation of otefin by PP2A-Tws promotes its interactions with BAF and lamin during NER. CDK1-dependent disruption of the Ote–BAF–lamin complex may act in concert with NHK-1/VRK1-dependent disruption of the BAF-DNA interactions and with the dissolution of the lamina during NEBD [95]. However, we found that otefin phosphorylation at Ser50 and/or Ser54 is not essential for oocyte development or embryogenesis, arguing against an essential role for this event. Nevertheless, our results suggest that once otefin is phosphorylated at Ser50 and Ser54, it must be dephosphorylated at these sites to be reincorporated in a complex with BAF and lamin and to be recruited to reassembling nuclei in a timely manner. Failure in this dephosphorylation can even be lethal for the embryo, albeit with incomplete penetrance. An analysis of the subcellular phenotypes developing in embryos expressing phosphomimetic otefin as a sole form of otefin was precluded by the difficulty in obtaining a large number of flies of the required genotype and by the difficulty to visualize the first mitoses by live imaging.

Apart from its roles in nuclear reassembly, otefin is required for female germline stem cell (GSC) differentiation and survival [81,96]. The expression of otefin in GSCs is also required for the survival of niche cells in a non-cell-autonomous manner [97]. In GSCs, the loss of otefin results in nuclear lamina defects and triggers cell death by a mechanism involving the ATR and Chk2 kinases [98]. The loss of BAF in GSCs results in similar defects [99]. Our results indicate that phosphorylation or dephosphorylation of otefin at Ser50–Ser54 is not strictly required for otefin essential functions in GSCs, nor are they required for the subsequent mitoses leading to the 16-cell cysts before oogenesis. The two otefin orthologues Bocks and MAN1 may help compensate for the misregulation of otefin in these germline cells. In somatic cells, complete loss of otefin does not cause any obvious phenotype but it is synthetic lethal in combination with mutation of *bocks* or *MAN1*, indicating that LEM-D proteins function semi-redundantly [80].

Our findings reveal the importance of establishing contact between BAF and a LEM-D protein of the INM during nuclear reassembly. Indeed, our results indicate that the restoration of the otefin–BAF interaction by the dephosphorylation of otefin at PP2A-Tws-dependent sites is essential during embryogenesis. We did not investigate if Bocks or MAN1 are regulated in their interactions with BAF by a similar mechanism. In addition, whether the NEBD-NER cycle relies on the phosphoregulation of BAF interactions with LEM-D proteins in other somatic cell types of *Drosophila* could be further explored.

In human cells, mitotic phosphorylation of Lem2 in the disordered region C-terminal to its LEM domain was shown to negatively regulate the ability of Lem2 to oligomerize into BAF-associated liquid condensates that interact with microtubules [100]. These structures reassemble in telophase and recruit the ESCRT-III complex for its essential membrane sealing activity during NER [101]. Thus, dephosphorylation of Lem2 during mitotic exit is thought to promote its essential recruitment at reassembling nuclei, although the responsible phosphatase is unknown [8,100]. While the recruitment of human emerin to reassembling nuclei was not yet reported to be regulated by phosphorylation, its ability to engage in homomeric interactions and to bind BAF is known to be influenced by a complex interplay between multisite phosphorylation and O-GlcNAcylation [102,103]. Thus, the phosphoregulation of the emerin–BAF interaction could play an important role in NER in human cells, promoting the initial recruitment of membranes to BAF-coated chromatin, before they can be sealed. Moreover, given that mutations in emerin and BAF result in progeria and laminopathies, defects in the regulation of their interaction could potentially contribute to human disease [104,105].

## 4. Materials and methods

### 4.1. Plasmids and mutagenesis

*Drosophila* cells expression vectors were generated by Gateway recombination (Invitrogen). Coding sequences were first cloned into the pDONR221 entry vector and then recombined into the relevant destination vectors for expression from copper-inducible (pMT) or constitutive (pAC5) promoters. The following expression vectors were generated: pAc5-Myc-BAF, pMT-GFP-Ote, pMT-GFP-Ote^AA^, pMT-GFP-Ote^DD^, pMT-GFP-Ote 1–70, pMT-GFP-Ote 42–400, pMT-GFP-Ote*Δ*TM, pMT-GFP-Ote^AA^*Δ*TM, pMT-GFP-Ote^DD^*Δ*TM and pAc5-mCherry-tubulin. *Drosophila* expression vectors were generated in the pUAS-K10 attB vector (UASp). The following were generated: pUAS-GFP-Ote^WT^, pUAS-GFP-Ote^AA^, pUAS-GFP-Ote^DD^, pUAS-GFP and pUAS-RFP-BAF. Amino acid substitution mutants were generated using QuikChange Lightning Site-Directed Mutagenesis Kit (Agilent) following the manufacturer's protocol.

### 4.2. Cell culture, transfections and cell lines

All cells were in the D-Mel (d.mel-2) background and were cultured in Express Five medium (Invitrogen) supplemented with glutamine, penicillin and streptomycin (Wisent). Transfections were performed using X-tremeGENE HP DNA Transfection Reagent (Roche) following the manufacturer's instructions. All stable cell lines were selected in medium containing 20 µg ml^−1^ blasticidin. While inducible pMT-based vectors contain the blasticidin resistance gene, pAc5-based vectors were co-transfected with pCoBlast to confer blasticidin resistance to the cells. Expression of the copper-inducible transgenes was induced with CuSO_4_ (300 µM unless otherwise indicated) for at least 8 h before experiments.

For RNA interference, dsRNAs were generated from PCR amplicons using a T7 RiboMAX kit (Promega). dsRNA derived from the bacterial kanamycin resistance gene was used as a non-target control. Twenty milligrams of dsRNA was transfected in 1X10^6^ cells in a well of a 6-well plate using Transfast transfection reagent according to the manufacturer's protocol.

### 4.3. Protein purifications for mass spectrometry

GFP affinity purifications from embryos destined to mass spectrometry analysis were done essentially as described [106]. Briefly, embryos were collected every 2 h and dechorionated in 50% bleach. For each genotype, 300 mg of embryos were crushed using a plastic pestle fitted to a 1.5 ml microfuge tube, in 1 volume of extraction buffer (20 mM Tris pH 7.5, 150 mM NaCl, 2 mM MgCl_2_, 0.5 mM EGTA, 1 mM DTT, 0.5% Triton X-100, 5% glycerol, 1 mM phenylmethylsulfonyl fluoride (PMSF), 10 µg ml^−1^ aprotinin and 10 µg ml^−1^ leupeptin). Additional extraction buffer was added to a total of 4.5 volumes. Lysates were passed four times through a needle using a syringe and were incubated on a rotating wheel at 4°C for 15 min. Samples were centrifugated at 21 000*g* at 4°C for 10 min. Supernatants were transferred to new tubes, avoiding the fat layer at the top. Samples were centrifugated again and supernatants were transferred again to new tubes. One hundred microliters of pre-equilibrated GFP-Trap agarose (Chromotek) was added to each sample. Samples were incubated on a rotating wheel at 4°C for 2 h. Beads were collected by centrifugation at 500 g at 4°C for 3 min. Supernatants were discarded. Beads were washed three times with 1 ml of extraction buffer, placing tubes on the wheel at 4°C for 5 min, and centrifugating them to collect beads. Samples were transferred to new tubes and four additional washed were done using final wash (20 mM Tris pH 7.5, 150 mM NaCl, 2 mM MgCl_2_, 0.5 mM EGTA and 1 mM DTT). Samples were transferred to new tubes again. Proteins were eluted by the addition of 750 µl of 1 M NH4OH, 0.5 mM EDTA, placing tubes on a rotating wheel at room temperature for 5 min. Eluates were transferred to new tubes. A second elution was done and pooled with the first one. Samples were split into two tubes: 90% destined to mass spectrometry analysis and 10% for visualization using electrophoresis. Samples were desiccated in a Speedvac.

### 4.4. Mass spectrometry

For phosphoproteomics, harvested cells were lysed in ice cold 1% (w/v) SDC (sodium deoxycholate, Sigma D6750) in 50 mM NH_4_HCO_3_. Protein concentration was measured by BCA assay (Thermo Fisher Scientific). Protein disulfide bonds were reduced by adding Tris(2-carboxyethyl)phosphine hydrochloride (TCEP, 5 mM final) to the lysates. Alkylation of cysteine residues was achieved by adding chloroacetamide (CAA, 20 mM final). Samples were incubated at 37°C with 500 rpm shaking for 30 min for the reduction/alkylation procedure and then digested (overnight, 37°C) with trypsin (Sigma-Aldrich) using an enzyme to substrate ratio of 1 : 25 (w:w). Tryptic digests were acidified with 1% formic acid (FA), centrifuged (17 000*g*, 10 min) and desalted on Oasis HLB cartridges (Waters) previously conditioned with acetonitrile (ACN) 1% FA, solid-phase extraction (SPE) buffer (50% ACN1% FA) and finally 1% aqueous FA. Peptide samples were applied, desalted with 3 ml of 1% FA and eluted in 1 ml of SPE buffer. Peptide eluates were snap-frozen in liquid nitrogen, lyophilized in a SpeedVac centrifuge and stored at −80°C. Phosphopeptide enrichment was performed on 5 µm titansphere particles (Canadian Life Science, Peterborough, ON, Canada) according to published protocols [107,108]. Loading of protein extracts on the titansphere beads, washing and elution steps were performed using custom spin columns [109] made from 200 µl pipette tip containing a SDB-XC membrane (Empore, 3 M) frit and filled with TiO_2_ beads. Peptides were desalted in 100 µl of 1% FA and subsequently eluted from spin columns using 100 µl of 50% ACN, 0.5% FA. Liquid chromatography-mass spectrometry (LC-MS)/MS analyses were performed on a Q-Exactive HF or an Orbitrap tribrid Fusion mass spectrometer using home-made capillary LC columns (18 cm length, 150 µm inner diameter and 360 µm outer diameter). Capillary LC columns were packed with C18 Jupiter 3 µm particles (Phenomenex, Torrance, CA) at 1000 psi. Samples were directly injected on LC-columns and separations were performed at a flow rate of 0.6 µl min^−1^ using a linear gradient of 5–35% aqueous ACN (0.2% FA) in 150 min. Raw data analysis of SILAC experiments was performed using Maxquant software 1.5.3.8. The false discovery rate (FDR) for peptide, protein and site identification was set to 1%; the minimum peptide length was set to 6. The Uniprot fly proteome database was used for all database searches. Further bioinformatics analyses were performed in R.

For mass spectrometry analysis of affinity purification products, eluted protein purification products were analysed by LC-MS as described [110]. Samples were reconstituted in 50 mM ammonium bicarbonate with 10 mM TCEP [Thermo Fisher Scientific] and vortexed for 1 h at 37°C. Chloroacetamide (Sigma-Aldrich) was added for alkylation to a final concentration of 55 mM. Samples were vortexed for another hour at 37°C. One microgram of trypsin was added, and digestion was performed for 8 h at 37°C. Supernatants were desalted on stage-tips (The Nest Group). Samples were dried down and solubilized in 5% ACN0.2% FA. The samples were loaded on a home-made reversed-phase column (150-μm i.d. by 150 mm) with a 220 min gradient from 10 to 30% ACN-0.2% FA and a 600-nl min^−1^ flow rate on an Easy nLC-1000 connected to an Orbitrap Fusion (Thermo Fisher Scientific, San Jose, CA). Each full MS spectrum acquired at a resolution of 240 000 was followed by tandem-MS (MS-MS) spectra acquisition on the most abundant multiply charged precursor ions for a maximum of 3 s. Tandem-MS experiments were performed using collision-induced dissociation at a collision energy of 30%. The data were processed using PEAKS X (Bioinformatics Solutions, Waterloo, ON) and a Uniprot *Drosophila* unreviewed database. Scaffold version 5.0.0 (Proteome Software Inc., Portland, OR) was used to validate MS/MS-based peptide and protein identifications.

### 4.5. Affinity co-purifications for western blots

For immunoprecipitation of Myc-BAF, pelleted cells from confluent 9 cm^2^ wells were lysed in 1 ml of lysis buffer (50 mM Tris-HCl pH 7.5, 150 mM NaCl, 1 mM EDTA, 10% glycerol, 0.2% Triton X-100, 1 mM PMSF, 10 µg ml^−1^ aprotinin and 10 µg ml^−1^ leupeptin), and lysates were centrifuged at 19 000*g* during 10 min at 4°C. Supernatants were incubated with anti-Myc 9E10 from mouse (#sc-40, Santa Cruz Biotechnology, Inc.) for 90 min at 4°C and then incubated with 20 µl of Protein G-conjugated Dynabeads (Invitrogen) for 30 min at 4°C, before being washed in lysis buffer four times.

For GFP affinity purifications, pelleted cells from confluent 25 cm^2^ flasks were lysed in 1 ml of lysis buffer (20 mM Tris-HCl pH 7.5, 150 mM NaCl, 2 mM MgCl_2_, 0.5 mM EDTA, 1 mM DTT, 5% glycerol, 0.5% NP40 Substitute, 1 mM PMSF, 10 µg ml^−1^ aprotinin and 10 µg ml^−1^ leupeptin). After 15 min on a rotating wheel at 4°C, lysates were centrifuged at 19 000xg during 10 min at 4°C. Supernatants were incubated with 20 µl of GFP-Trap agarose (Chromotek) for 2 h at 4°C, before being washed four times in wash buffer (20 mM Tris-HCl pH 7.5, 150 mM NaCl, 2 mM MgCl_2_, 0.5 mM EDTA, 1 mM DTT, 5% glycerol, 0.1% NP40 Substitute, 1 mM PMSF, 10 µg ml^−1^ aprotinin and 10 µg ml^−1^ leupeptin).

### 4.6. *Drosophila* genetics

Fly husbandry was conducted according to standard procedures. All crosses were performed at 25°C. The WT strain used was Oregon R. Transgenic lines for expression of *UASp-GFP-Ote* (WT and mutants) were generated by site-directed insertions of our pUAS-K10attB-based vectors on the third chromosome in the attP154 strain (BestGene). The expression of the *UASp-GFP-Ote* and *UASp-RFP-BAF* transgenes in embryos for GFP affinity purification and for video microscopy was driven by *matα4-GAL-VP16* (#7062; Bloomington *Drosophila* Stock Center). For genetic rescue experiments, the expression of *UASp-GFP-Ote* transgenes was driven by *GAL4::VP16-nos.UTR* (MVD1) (no. 4937; Bloomington *Drosophila* Stock Center). *Otefin* mutant alleles used were *ote^B279^* (no. 16189; Bloomington *Drosophila* Stock Center) and *ote^DB^* (no. 5092; Bloomington *Drosophila* Stock Center). Fertility tests were done by placing single females with 2–3 Oregon R males in tubes containing grape juice agar and yeast paste at 25°C. Flies were transferred to new tubes every day and the number of eggs laid and percentages of hatched embryos were counted 24 h after removal of the flies from the tubes.

### 4.7. Western blotting

Primary antibodies used in western blotting were anti-GFP from rabbit (no. A6455 at 1 : 5000; Invitrogen), anti-Myc 9E10 from mouse (no. sc-40 at 1 : 1000 dilution for WB; Santa Cruz Biotechnology), anti-lamin Dm0 from mouse (Developmental Studies Hybridoma Bank Hybridoma Products ADL84.12 deposited by P. A. Fisher, at 1 : 500) and anti-BAF (custom made by Thermo Scientific at 1 : 1000). Secondary antibodies were coupled to peroxidase (1 : 5000 dilution; Jackson ImmunoResearch). All antibodies were diluted in tris-buffer saline with 0.1% Tween 20 and 5% dry milk.

### 4.8. Microscopy

Live imaging was performed using a spinning-disc confocal system (Yokogawa CSU-X1 5000) mounted on a fluorescence microscope (Zeiss Axio Observer Z1) using an Axiocam 506 mono camera (Zeiss), 63× oil objective (NA 1.4) and ZEN software. For time-lapse microscopy of D-Mel cells, cells in culture were plated in a Lab-Tek II chambered coverglass (no. 155409; Thermo Fisher Scientific). For live analysis of *Drosophila* syncytial embryos, 0 to 2 h old embryos were first dechorionated in 50% bleach, aligned on a coverslip (no. P35G-1.5-14-C; MatTek) and covered with halocarbon oil. Between several confocal sections at 1 µm spacing were collected at each time point.

Embryo injection was performed using home-made glass capillary fixed on an electronically controlled micromanipulator installed on the microscope. Injections were triggered manually using an air-filled plastic syringe connected to the capillary through a rubber tube. All fluorescence quantifications and images treatment were performed using ZEN software (Zeiss).

For quantifications of fluorescence in embryos, measurements were made from the hand-drawn inner core area from a single Z-step intersecting the nucleus at each time point. Time zero was set as the time preceding the onset of RFP-BAF recruitment. Fluorescence measured inside the nuclear area at time zero was subtracted from all values. For quantifications of fluorescence in cells, measurements were made from maximum-intensity projections from Z-stacks taken with a 1 µm interspace. Measurements were taken from circular areas corresponding to the approximate positions of reassembling nuclei. Fluorescence measured inside the cytoplasm at each time point was subtracted from all values. As cells differed in their expression levels, values for each time point were normalized, setting the time zero value to one for each cell.

### 4.9. Statistical analysis

Statistical analyses of the fluorescence, Western blots and genetic data were done using GraphPad, using tests indicated in the figure legends. Statistical analyses of the mass spectrometry data were done using Perseus [111] and data visualized with Origin Pro software (OriginLab Corp.).

## Data Availability

Data have been deposited to the ProteomeXchange Consortium via the PRIDE partner repository with the dataset identifiers PXD041030 and PXD041032. Additional information is provided in the electronic supplementary material [112].
